# Impact of combined consumption of fish oil and probiotics on the serum metabolome in pregnant women with overweight or obesity

**DOI:** 10.1016/j.ebiom.2021.103655

**Published:** 2021-10-30

**Authors:** Kati Mokkala, Tero Vahlberg, Noora Houttu, Ella Koivuniemi, Leo Lahti, Kirsi Laitinen

**Affiliations:** a Institute of Biomedicine, Integrative Physiology and Pharmacology, 20014 University of Turku, Turku, Finland; b Institute of Clinical Medicine, Biostatistics, 20014 University of Turku, Turku, Finland; c Department of Computing, 20014 University of Turku, Turku, Finland; d Department of Obstetrics and Gynecology, Turku University Hospital, Kiinamyllynkatu 4-8, 20520 Turku, Finland

**Keywords:** Fish oil, Probiotics, Metabolomics, Gestational diabetes, Intervention, DHA, docosahexaenoic acid, DPA, docosapentaenoic, EPA, eicosapentaenoic acid, GDM, gestational diabetes, BH, Benjamini-Hochberg, BMI, body mass index, FDR, false discovery rate, hsCRP, high sensitivity C-reactive protein, IQR, interquartile range, glycoprotein acetylation, GlycA, MUFA, monounsaturated fatty acids, OGTT, oral glucose tolerance test, PUFA, polyunsaturated fatty acids

## Abstract

**Background:**

If a pregnant woman is overweight, this can evoke metabolic alterations that may have health consequences for both mother and child.

**Methods:**

Pregnant women with overweight/obesity (*n* = 358) received fish oil+placebo, probiotics+placebo, fish oil+probiotics or placebo+placebo from early pregnancy onwards. The serum metabolome was analysed from fasting samples with a targeted NMR-approach in early and late pregnancy. GDM was diagnosed by OGTT.

**Findings:**

The intervention changed the metabolic profile of the women, but the effect was influenced by their GDM status. In women without GDM, the changes in nine lipids (FDR<0.05) in the fish oil+placebo-group differed when compared to the placebo+placebo-group. The combination of fish oil and probiotics induced changes in more metabolites, 46 of the lipid metabolites differed in women without GDM when compared to placebo+placebo-group; these included reduced increases in the concentrations and lipid constituents of VLDL-particles and less pronounced alterations in the ratios of various lipids in several lipoproteins. In women with GDM, no differences were detected in the changes of any metabolites due to any of the interventions when compared to the placebo+placebo-group (FDR<0.05).

**Interpretation:**

Fish oil and particularly the combination of fish oil and probiotics modified serum lipids in pregnant women with overweight or obesity, while no such effects were seen with probiotics alone. The effects were most evident in the lipid contents of VLDL and LDL only in women without GDM.

**Funding:**

State Research Funding for university-level health research in the Turku University Hospital Expert Responsibility Area, Academy of Finland, the Diabetes Research Foundation, the Juho Vainio Foundation, Janssen Research & Development, LLC.

**Research in context**

**Evidence before this study**

Before the initiation of this study trial, in 2013, we searched PubMed and ClinicalTrials.gov using the terms “fish oil”, “probiotics”, “gestational diabetes”, metabolomics”. It seemed that new metabolomics methodology had been rarely applied in this kind of study. Further, no trials combining two commonly used dietary supplements, fish oil and probiotics, had been conducted or were planned. Ingredients in both supplements have properties that could beneficially modify the serum metabolome. During the time between the initiation of the study and the submission of this manuscript, no trials investigating the impact of the combination of these two supplements on maternal metabolomics have been identified.

**Added value of this study**

The supplementation of the combination of fish oil and probiotics induced more changes in the metabolites, than seen with supplementation with fish oil only. These alterations were dependent on whether or not the women developed gestational diabetes (GDM): the changes were detected only in women without GDM.

**Implications of all the available evidence**

Most of the changes induced by the intervention are considered to be of benefit in terms of the long-term risk for combatting the metabolic disturbances associated with overweight and obesity. The study provides important insights into potential means of modifying the metabolic profile in a population at high risk for developing metabolic disturbances.

Alt-text: Unlabelled box

## 1 Introduction

Maternal lipid and carbohydrate metabolism undergoes several alterations throughout the course of pregnancy [1]. These physiological changes are normally tightly regulated, but aberrations, e.g. due to maternal obesity, may predispose both the mother and her child to health complications, one example being gestational diabetes (GDM) in the mother and macrosomia in the child [2]. As obesity is increasingly encountered in reproductive age women [3], novel approaches are necessary to mitigate the detrimental effects of overweight and obesity on maternal metabolism. Indeed, the maternal metabolome associates with BMI [4], and overweight and obese women have been demonstrated to exhibit a distinct serum metabolic profile from that of normal weight women [5,6]. These findings highlight the need to search for effective interventions to minimize the aberrant metabolism occurring during pregnancy.

Traditional means to modify the metabolic health in a high risk population include lifestyle changes, such as a dietary modification. Two dietary supplements, probiotics and fish oil are of interest as both have been shown to exert health benefits e.g. by modulating multiple metabolic events like the regulation of glucose and insulin metabolism [7], and reducing the level of low grade inflammation [8], However, there is no consensus of their putative benefits in modulating maternal health during pregnancy [9,10], but novel metabolic markers, such as those revealed by a metabolomics analysis, could provide insights into the ways in which they may modify metabolism.

In contrast to the traditional metabolic markers e.g. serum levels of cholesterol and triglycerides, a metabolomic analysis gathers information on the abundance of metabolites and thus provides a comprehensive view of the metabolic profile. This was evident in our previous study in which we observed that the metabolic profile of women who were developing GDM differed already in early pregnancy from those who would remain healthy [11]. Others have demonstrated links between the maternal serum metabolome and excessive gestational weight gain [6], term preeclampsia [12], fetal growth restriction [13] and spontaneous preterm birth [5] or stillbirth [14]. Previous data using metabolomics in non-pregnant adults revealed some benefits of either probiotics or fish oil on metabolism, particularly on lipid metabolism [15], [16], [17], [18]. To our knowledge, there are no studies which would have investigated the influence of probiotics and fish oil on serum metabolomics of pregnant women with overweight or obesity, a high risk group for developing metabolic diseases, let alone studies investigating the impact of a combination of these supplements on overall metabolomics.

As those women with overweight and obesity are at high risk for metabolic disorders including GDM, we wanted to investigate whether supplementation of probiotics and fish oil would benefit the metabolic profile of this vulnerable population. Thus, our first aim was to investigate the effects of supplementing diet with probiotics and fish oil, either separately or in combination, compared to placebo, to modify serum metabolites, as analysed using metabolomics over the course of pregnancy in pregnant women with overweight or obesity. Secondly, as the metabolic profile is disturbed in GDM [19], we investigated whether women with or without GDM would respond differentially to the intervention.

## 2 Materials and methods

### 2.1 Participants and design

Serum metabolomic profiles were analysed in women participating in a mother-infant dietary single-center intervention trial (ClinicalTrials.gov, NCT01922791) being conducted in Southwest Finland. The study protocol has been described in detail previously in the ClinicalTrials.gov and in Pellonperä et al. [9]. Briefly, the inclusion criteria for the study were overweight (prepregnancy BMI ≥25) and early pregnancy (<18 weeks of gestation). The exclusion criteria were GDM diagnosed before the first study visit, multifetal pregnancy, the presence of metabolic or inflammatory diseases. Serum samples were available from 358 of these women to allow us to conduct a metabolomic analysis in early (at gestational weeks median 14•1 (IQR 12•7-15•4)) and the late pregnancy (at gestational weeks 35•1 (34•6-35•9)) (Supplemental figure 1). Prepregnancy BMI (kg/m^2^) was calculated by dividing self-reported weight in kilograms, obtained from women's welfare clinic records, by height measured with a wall stadiometer to the nearest 0•1 cm in early pregnancy. The characteristics of the women (Table 1), including age, education, GDM in a previous pregnancy, smoking and a diagnosis of diabetes or metabolic syndrome in the mother's parents, were collected in the questionnaires.

**Table 1:** Baseline characteristics of the women.

	Fish oil+placebo *n* = 88	Probiotics+placebo *n* = 91	Fish oil+probiotics *n* = 90	Placebo+placebo *n* = 89	All*n* = 358	*P*-value
Age (years)	31.0 (26.6-34.0)	31 (28-35)	30 (28-34)	30 (28-33)	30 (28-34)	0.832
Prepregnancy BMI	29.2 (27.0-32.8)	28.0 (26.5-30.8)	28.4 (25.8-31.6)	29.3 (26.5-32.1)	28.7 (26.5-31.9)	0.237
Obese	46.6% (41/88)	33.0 % (30/91)	37.8% (34/90)	41.6% (37/89)	39.7% (142/358)	0.291
Gestational weeks at 1. visit	14.3 (13.0-15.7)	13.9 (12.4-15.3)	14.3 (12.9-15.2)	14.1 (12.7-15.4)	14.1 (12.7-15.4)	0.701
Gestational weeks at 2. visit	35.1 (34.6-35.7)	35.3 (34.6-36.0)	35.1 (34.6-35.7)	35.1 (34.6-36.0)	35.1 (34.6-35.9)	0.895
Gestational weeks at early pregnancy OGTT	14.9 (13.0-16.3)	14.7 (12.9-15.7)	15.3 (13.9-16.2)	14.0 (12.6-15.4)	14.7 (13.0-16.0)	0.211
Gestational weeks at mid-pregnancy OGTT	26.3 (25.1-27.4)	25.9 (25.1-26.7)	26.0 (25.0-27.9)	25.8 (25.0-26.9)	25.9 (25.0-27.3)	0.418
GDM diagnosis at mid-pregnancy	20.5% (16/78)	24.7% (21/85)	25.9% (21/81)	19.0% (15/79)	22.6% (73/323)	0.680
Highly educated	69.3% (66/88)	65.5% (57/87)	58.4% (52/89)	58.6% (51/87)	63.0% (221/351)	0.354
Smoked during pregnancy	2.3% (2/88)	6.8% (6/88)	4.5% (4/88)	6.9% (6/87)	5.1% (18/351)	0.455
Smoked before pregnancy	17.0% (15/88)	27.0% (24/89)	12.4% (11/89)	28.7% (25/87)	21.2% (75/353)	0.020

Values are medians (IQR) or percentages. The differences in maternal characteristics were analyzed using chi-square-test for categorical variables and Kruskall-Wallis-test for continuous variables.

The probiotic capsules contained *Lactobacillus rhamnosus* HN001 (ATCC SD5675; DuPont, Niebüll, Germany) and *Bifidobacterium animalis ssp. lactis* 420 (DSM 22089; DuPont), each 10^10^ colony-forming units per capsule. The placebo for the probiotics contained microcrystalline cellulose; the capsules were identical to the probiotic capsules in size, shape, and color. The capsules were stored at −20°C until provided to the subjects, who were instructed to store the capsules in a refrigerator. Fish oil supplement capsules (Croda Europe Ltd., Leek, U.K) contained 2•4 g of n-3 fatty acids, 1•9 g docosahexaenoic acid (22:6 n-3) DHA and 0•22 g eicosapentaenoic acid (20:5 n-3) EPA and 0•28 g other n-3 fatty acids, such as docosapentaenoic acid (DPA). The placebo capsules for the fish oil contained medium-chain fatty acids (capric acid C8 54•6%, caprylic acid C10 40•3 %). *L. rhamnosus HN001* is a well characterized probiotic [20] and *B. animalis ssp. lactis 420* is a novel probiotic with demonstrated health benefits related to metabolism in an animal study [21] and inflammation in humans [22,23]. Long chain PUFA, in this case fish oil, which is rich in DHA and EPA, are known inflammation-resolving dietary factors [24] and are important for fetal and child development [25], and may possibly reduce insulin resistance [26]. The dose chosen for fish oil was considered to be safe and to yield benefits for both mother and child [27]. The stability of the supplements was monitored by both manufacturers regularly during the trial.

**Fig. 1:**
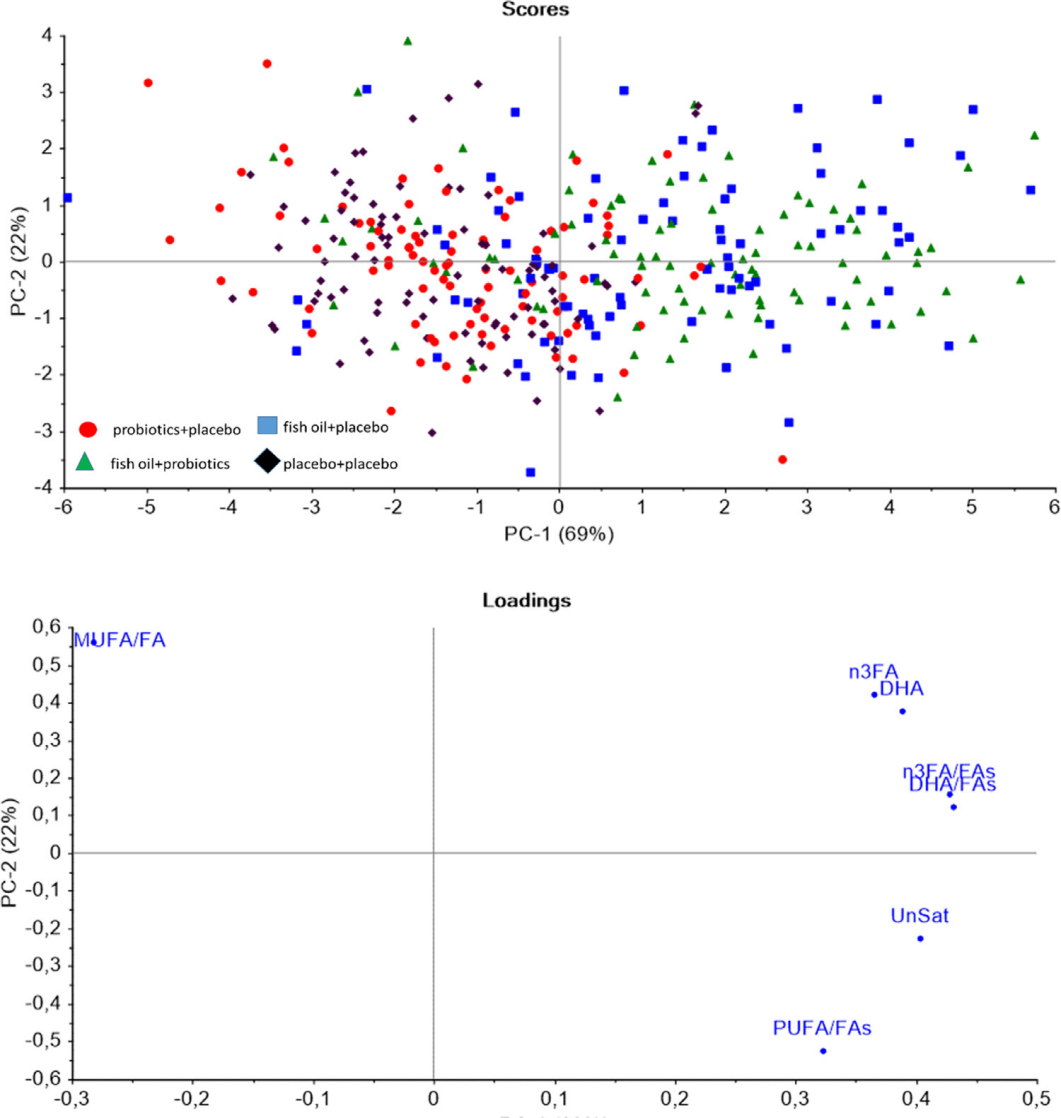
PCA of the lipids that reflect the intake of fish oil. All women included. Blue= fish oil+placebo, red= probiotics+placebo, green= fish oil+probiotics, black= placebo+placebo.

The women were randomized in a double-blind manner into four intervention groups (Table 1): fish oil+placebo, probiotics+placebo, fish oil+probiotics or placebo+placebo from the first study visit throughout the pregnancy, and until 6 months postpartum. The compliance with the intervention was reported as good by 88•4% of the women and when calculated from the returned fish oil capsules, a mean of 91•8% (SD 15.9) of the capsules had been consumed [9]. The compliance was similar in both GDM groups (women without GDM 89•7% and with GDM 83•5% with good compliance). Good compliance was confirmed in PCA, which revealed a clear separation of the intervention groups according to lipids that reflected the intake of fish oil (Fig. 1). A complementary, supervised Partial Least Squares (PLS) discriminant analysis for the selected lipid species had a mean error rate of 17.9% (std 4%) in 5-fold cross-validation; the random expectation being 51%. The PLS scores and loadings are included in the supplementary material (Supplemental figure 2 and 3). Adverse effects (gastrointestinal symptoms or headache) of the capsule consumption were reported by 28% of the women, with no significant differences among the intervention groups as reported previously [9]

### 2.2 Ethics

This study was conducted according to the guidelines laid down in the Declaration of Helsinki as revised in 2013; all procedures that involved human subjects were approved by the Ethics Committee of the Hospital District of Southwest Finland (permission number 115/180/2012) and all participants provided written informed consent.

### 2.3 Outcomes of the study

In this part of the trial, the principal aims were to investigate the impact of the intervention on serum metabolomics and to examine whether the response had differed in women with or without GDM. These were predefined secondary outcomes of the main study (the primary outcome has been reported earlier) [9].

### 2.4 Blood sampling and analytical methods

Fasting (9 h minimum) blood samples were drawn from the antecubital vein, and the serum was separated and frozen in aliquots at -80°C until being analyzed by serum metabolomics. A high-throughput proton NMR metabolomics platform (Nightingale, Helsinki, Finland) was used to analyze the serum metabolic profile as described earlier [28]. The biomarker platform comprises 228 metabolites and their ratios, including biomarkers of lipid and glucose metabolism, amino acids, ketone bodies and glycoprotein acetylation (GlycA), a marker of low grade inflammation. GlycA consists of a complex heterogeneous nuclear magnetic resonance signal originating from the N-acetyl sugar groups on multiple acute phase glycoproteins present in the circulation; α1-acid glycoprotein, haptoglobin, α1-antitrypsin, α1-antichymotrypsin and transferrin [29].

GDM was diagnosed in mid-pregnancy (gestational weeks 25•9 (25•0-27•3)) according to national guidelines, as previously described [9]. Those women who were diagnosed with GDM in early pregnancy, were not included when analyzing the interaction of GDM status and the intervention.

### 2.5 Statistics

The normality of the distributions of the data was analyzed by visual inspection of histograms and as most of the variables were not normally distributed (skewness >1), we used non-parametric tests. Several subjects had zero values in extremely large, extra-large and large VLDL particles, and the values of these variables were excluded from the analysis according to the instructions from the analyser. In early pregnancy, no differences between the intervention groups were detected in any of the metabolites. When the differences in the changes from early to late pregnancy (total 228 variables) were analyzed between the intervention groups, we applied Kruskall-Wallis-test, followed by pairwise comparisons using Mann-Whitney U-test with Bonferroni corrections. When investigating the change in serum metabolomics according to the women's GDM status, those women with early GDM diagnoses were excluded, while when analyzing the differences between metabolites in late pregnancy according to their GDM status, both early and late GDM diagnoses were included in the analyses. To study the pregnancy induced changes in the metabolites, we conducted Wilcoxon signed ranked test to analyze the change from early to late pregnancy in the placebo+placebo group. The metabolomic variables were adjusted for multiple comparison using the Benjamini-Hochberg procedure (BH-procedure), the false discovery rate (FDR) with <0•05 considered as a statistically significant threshold. To transform the skewed metabolomics variables so that they followed a normal distribution, we used a rank-based inverse normal transformation with Blom's method. Normal scores were used in two-way analysis of variance when investigating the interaction between GDM status and the intervention. Normal scores were also used in the figures in order to ensure that the metabolomics variables were comparable with each other.

The differences in maternal characteristics were analyzed using chi-square-test for categorical variables and Kruskall-Wallis-test for continuous variables. Smoking before pregnancy differed between the intervention groups (Table 1). However, no differences were detected in the metabolites between women who smoked before pregnancy compared to those who did not (Mann-Whitney U-test corrected with BH-procedure, data not shown), thus analyses were not adjusted for smoking status before pregnancy.

These analyses were performed using SPSS version 25 (IBM Inc.). Principal component analysis (PCA) (Unscrumble, CAMO, country, etc.) including all of the four intervention groups was conducted to evaluate the impact of fish oil intake on those lipids that would be expected to change due to the intake of fish oil and thus would reflect also the compliance to food supplements. Further, we carried out Partial Least Squares (PLS) discriminant analysis as a supervised method to assess the ability of the aforementioned lipid species to discriminate between intervention groups. The lipid abundances were scaled to zero mean and unit variance before the analysis, and we tested the ability to discriminate the subjects who received fish oil (fish oil+placebo and fish oil+probiotics groups) from the other subjects who did not receive fish oil (probiotics+placebo and placebo+placebo groups). The analysis was based on the mixOmics R package (version 6.17.26) function plsda with default settings. Furthermore, PCA was used to demonstrate the different responses to fish oil according to the GDM status. The quality of the PCA analyses was confirmed by checking the calibration and validation variances. The workflow of the study is presented as supplemental material (Supplemental figure 4).

The pre-specified outcomes of the study were serum metabolites, but at the time when the study was planned, there were no a priori data for the effects of probiotics or fish oil on serum metabolites during pregnancy, the secondary outcomes of the trial, thus power calculations for these outcomes could not be performed.

### 2.6 Role of Funders

This clinical trial was supported by the State Research Funding for university-level health research in the Turku University Hospital Expert Responsibility Area, Academy of Finland (#258606), the Diabetes Research Foundation and the Juho Vainio Foundation. Funding to the University of Turku for the metabolomics analyses and reporting was provided by Janssen Research & Development, LLC. LL was supported by Academy of Finland (#295741). The funding sources had no role in the design, execution, analyses, interpretation of the data, or decision to submit these results.

## 3 Results

### 3.1 Baseline characteristics and pregnancy induced alterations in metabolites

The prepregnancy BMI of all women was median 28•7 (IQR 26•5-31•9), 39•7% (142/358) being obese (BMI≥30). Altogether 22•6% of the women (73 out of 323 tested) were diagnosed with GDM (Table 1). When we evaluated only changes in the placebo+placebo group, which represents the change induced by pregnancy, 153 metabolites (out of 228) increased and 52 decreased, one remained constant (mean diameter of LDL-particles), throughout the course of the pregnancy (FDR<0•05, Wilcoxon). With respect to the lipids, the concentrations of several lipoproteins and their lipid contents increased, whereas some medium and large- sized HDL-particles with their constituents decreased. Furthermore, a decrease in total cholesterol in HDL and HDL2 was seen (Supplemental table 1). Increases were detected in the levels of lactate and pyruvate, markers of glucose metabolism, the ketone body acetoacetate, amino acids, i.e. in glycine, histidine, isoleucine, leucine and phenylalanine, while the levels of valine, glutamine and tyrosine declined. In addition, the concentration of glucose decreased while the marker for low grade inflammation, GlycA, increased.

### 3.2 Impact of intervention on serum metabolomics

**Fig. 2:**
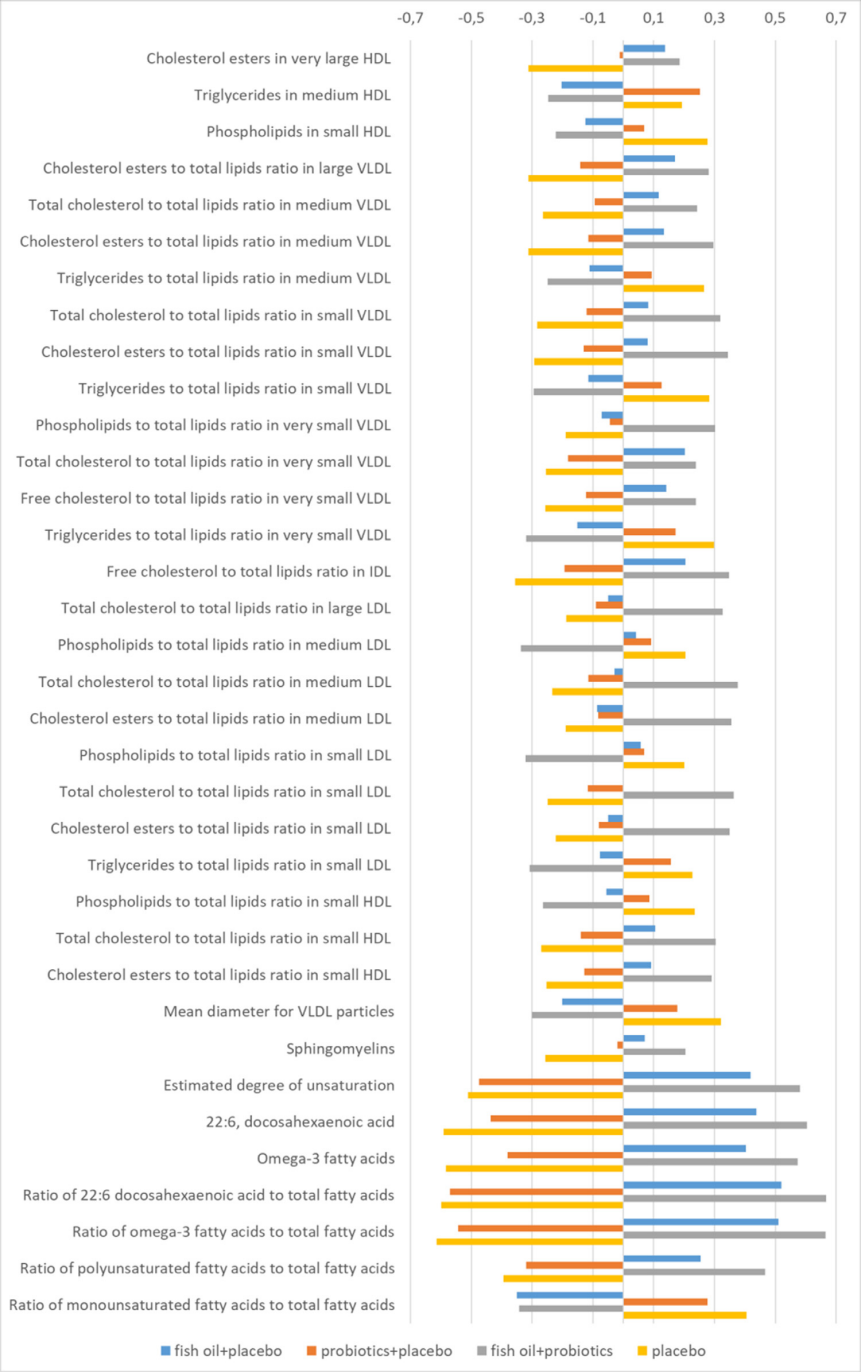
Metabolites (*n* = 35) with statistically significant differences in the changes between the fish oil+probiotics group and the placebo+placebo group. The figure shows the effect size of the variables when compared to each other. The lines represent normal scores obtained from rank-based inverse normal transformation by Blom's method. The mean of each variable is zero and thus a negative value indicates a change that is smaller than the mean change.

When comparing the change from early to late pregnancy, several differences in lipids, but not in other metabolites, were detected in the four intervention groups (FDR<0•05, Kruskall-Wallis-test). The most evident changes were detected in the fish oil+probiotics-combination group: 35 out of 228 metabolites differed when compared to placebo+placebo-group (FDR<0•05, Mann-Whitney U-test with post hoc Bonferroni corrections) (Table 2, Fig. 2, Supplemental figure 5). These were attributable to the concentrations of triglycerides in medium HDL-particles and mean diameter of VLDL-particles which increased less, cholesterol esters in very large HDL-particles and sphigomyelins which increased more and phospholipids in small HDL-particles which decreased more in the fish oil+probiotics- group as compared to the placebo+placebo-group. Most (23 out of 35) of the detected differences took place in the ratios of various lipids to total fatty acids in VLDL-, LDL-, IDL- and HDL-particles. In addition, as expected, significant changes were observed in lipid variables that reflected the intake of fish oil when comparing the fish oil+probiotics to placebo+placebo group (Table 2, Supplemental table 1).

**Table 2:** a. Statistically significant differences in the change of metabolites from early to late pregnancy between fish oil+probiotics group and placebo. The values are expressed as median (IQR) of the difference between early and late pregnancy concentrations.

	Fish oil+probiotics (*n* = 90)	Placebo+placebo (*n* = 89)	Fish oil+probiotics vs placebo+placebo
	Median (IQR)	Median (IQR)	BH-adjusted *P*-value*
Cholesterol esters to total lipids ratio in large VLDL	-6,85E-01 (-1,88E+00-1,40E-01)	-1,74E+00 (-3,07E+00–4,95E-01)	2,02E-02
Total cholesterol to total lipids ratio in medium VLDL	1,70E+00 (4,50E-02-3,74E+00)	2,50E-01 (-1,29E+00-2,19E+00)	2,87E-02
Cholesterol esters to total lipids ratio in medium VLDL	2,75E-01 (-1,25E+00-1,84E+00)	-9,50E-01 (-2,71E+00-5,15E-01)	7,00E-03
Triglycerides to total lipids ratio in medium VLDL	-1,92E+00 (-3,81E+00-2,03E-01)	-1,00E-01 (-2,23E+00-1,69E+00)	2,64E-02
Total cholesterol to total lipids ratio in small VLDL	2,29E+00 (5,20E-01-4,50E+00)	3,30E-01 (-1,95E+00-2,25E+00)	3,31E-03
Cholesterol esters to total lipids ratio in small VLDL	1,75E+00 (1,00E-01-3,55E+00)	-1,50E-01 (-2,21E+00-1,40E+00)	2,12E-03
Triglycerides to total lipids ratio in small VLDL	-6,80E-01 (-3,01E+00-1,24E+00)	1,43E+00 (-7,95E-01-3,47E+00)	6,26E-03
Phospholipids to total lipids ratio in very small VLDL	-1,20E-01 (-8,75E-01-4,38E-01)	-7,70E-01 (-1,59E+00–6,50E-02)	2,00E-02
Total cholesterol to total lipids ratio in very small VLDL	-1,90E+00 (-3,96E+00–4,40E-01)	-3,38E+00 (-5,27E+00–2,07E+00)	2,02E-02
Free cholesterol to total lipids ratio in very small VLDL	7,50E-02 (-2,93E-01-4,50E-01)	-2,50E-01 (-6,70E-01-2,30E-01)	2,38E-02
Triglycerides to total lipids ratio in very small VLDL	1,75E+00 (2,45E-01-4,93E+00)	4,40E+00 (2,64E+00-5,95E+00)	3,31E-03
Free cholesterol to total lipids ratio in IDL	-5,20E-01 (-1,03E+00–1,08E-01)	-1,26E+00 (-1,79E+00–6,45E-01)	1,50E-04
Total cholesterol to total lipids ratio in large LDL	1,01E+00 (-3,30E-01-2,01E+00)	-1,00E-01 (-1,23E+00-8,85E-01)	1,39E-02
Phospholipids to total lipids ratio in medium LDL	-3,70E+00 (-4,84E+00–2,23E+00)	-2,21E+00 (-3,89E+00–1,05E+00)	6,21E-03
Total cholesterol to total lipids ratio in medium LDL	2,77E+00 (9,05E-01-4,20E+00)	5,20E-01 (-1,16E+00-2,43E+00)	8,51E-04
Cholesterol esters to total lipids ratio in medium LDL	5,19E+00 (3,27E+00-7,52E+00)	3,16E+00 (7,55E-01-5,79E+00)	3,31E-03
Phospholipids to total lipids ratio in small LDL	-4,77E+00 (-6,47E+00–3,05E+00)	-2,98E+00 (-5,23E+00–1,41E+00)	1,31E-02
Total cholesterol to total lipids ratio in small LDL	3,26E+00 (1,43E+00-5,19E+00)	8,50E-01 (-1,34E+00-3,12E+00)	9,46E-04
Cholesterol esters to total lipids ratio in small LDL	5,31E+00 (3,08E+00-7,83E+00)	2,50E+00 (-1,30E-01-5,97E+00)	2,71E-03
Triglycerides to total lipids ratio in small LDL	1,39E+00 (2,65E-01-2,46E+00)	2,37E+00 (1,37E+00-3,26E+00)	1,81E-02
Phospholipids to total lipids ratio in small HDL	-4,67E+00 (-6,60E+00–2,81E+00)	-2,66E+00 (-5,10E+00–6,70E-01)	1,47E-02
Total cholesterol to total lipids ratio in small HDL	2,39E+00 (6,33E-01-4,48E+00)	1,40E-01 (-2,23E+00-2,57E+00)	3,31E-03
Cholesterol esters to total lipids ratio in small HDL	2,74E+00 (5,28E-01-5,09E+00)	3,80E-01 (-2,34E+00-3,08E+00)	6,26E-03

*Mann-Whitney U-test

**Table 2:** b. Statistically significant differences in the change of metabolites from early to late pregnancy between fish oil+probiotics group and placebo. The values are expressed as median (IQR) of the difference between early and late pregnancy concentrations.

	Fish oil+probiotics (*n* = 90)	Placebo+placebo (*n* = 89)	Fish oil+probiotics vs placebo+placebo
	Median (IQR)	Median (IQR)	BH-adjusted *P*-value*
Cholesterol esters in very large HDL mmol/l	4,93E-02 (8,93E-03-7,44E-02)	2,35E-02 (-1,12E-02-4,83E-02)	2,87E-02
Triglycerides in medium HDL mmol/l	1,47E-02 (8,99E-03-1,99E-02)	1,85E-02 (1,35E-02-2,69E-02)	3,71E-02
Phospholipids in small HDL mmo/l	-4,10E-02 (-8,43E-02–1,21E-02)	-2,00E-02 (-5,55E-02-2,74E-02)	3,27E-02
Mean diameter for VLDL particles nm	5,45E-01 (1,03E-01-1,11E+00)	1,11E+00 (5,65E-01-1,89E+00)	2,12E-03
Sphingomyelins mmol/l	9,12E-02 (5,52E-02-1,36E-01)	5,96E-02 (3,37E-02-1,13E-01)	4,35E-02
Estimated degree of unsaturation	-1,30E-02 (-4,45E-02-4,50E-03)	-6,50E-02 (-9,75E-02–4,00E-02)	6,73E-11
22:6, docosahexaenoic acid mmol/l	9,53E-02 (6,63E-02-1,32E-01)	2,48E-02 (1,28E-02-4,93E-02)	5,36E-15
Omega-3 fatty acids mmol/l	2,79E-01 (2,17E-01-3,73E-01)	1,10E-01 (5,69E-02-1,71E-01)	3,83E-14
Ratio of 22:6 docosahexaenoic acid to total fatty acids	2,10E-01 (-1,78E-02-4,28E-01)	-2,48E-01 (-3,72E-01–1,36E-01)	3,55E-15
Ratio of omega-3 fatty acids to total fatty acids	5,62E-01 (4,05E-02-1,10E+00)	-4,96E-01 (-9,06E-01–2,58E-01)	3,55E-15
Ratio of polyunsaturated fatty acids to total fatty acids	-2,41E+00 (-4,00E+00–1,24E+00)	-4,53E+00 (-5,77E+00–2,97E+00)	3,14E-06
Ratio of monounsaturated fatty acids to total fatty acids	2,12E+00 (1,15E+00-3,17E+00)	3,35E+00 (2,52E+00-4,39E+00)	6,56E-05

*Mann-Whitney U-test

In the fish oil+placebo group, nine metabolites differed when compared to the placebo+placebo group. In addition to the lipids associated with the intake of fish oil (as in the fish oil+probiotics group), the ratio of triglycerides to total lipids in very small VLDL-particles increased less and the ratio of free cholesterol to total lipids in IDL-particles decreased less when compared to the placebo+placebo group (Supplemental table 1).

No differences in any of the metabolites were observed when the women in probiotics+placebo-group were compared to the placebo+placebo group (Supplemental table 1).

### 3.3 Interaction between GDM status and the intervention

To reveal whether the GDM status of the women would influence the metabolic response to the intervention, we investigated separately women with or without GDM. Interestingly, the metabolic responses to the intervention were observed only in the women without GDM with no response being detected in any of the intervention groups in women with GDM (Supplemental table 2). In women without GDM, most of the differences were detected in the the fish oil+probiotics combination, followed by the fish oil+placebo group, while no differences were found in the probiotics+placebo group when compared to the placebo+placebo group. Firstly, in the fish oil+probiotics group, altogether 46 metabolites differed (FDR<0•05, Mann-Whitney U-test with post hoc Bonferroni corrections), these included the concentrations of VLDL-particles, their constituents and mean diameter, which increased less in the fish oil+probiotics-group when compared to the placebo+placebo group (Table 3). In addition, differences were observed in the ratios of several lipids to total fatty acids and the lipids reflecting the intake of fish oil (Table 3).

**Table 3:** a. Statistically significant differences in the change of metabolites from early to late pregnancy between the fish oil+probiotics group and the placebo+placebo in women without GDM. The values are expressed as median (IQR) concentration for the difference between early and late pregnancy.

	Fish oil+probiotics (*n* = 60)	Placebo+placebo (*n* = 64)	Fish oil+probiotics vs placebo+placebo
	Median (IQR)	Median (IQR)	BH-adjusted *P*-value*
Concentration of chylomicrons and extremely large VLDL particles mol/l	5,91E-11 (2,94E-11-1,00E-10)	1,17E-10 (6,63E-11-1,97E-10)	4,33E-02
Total lipids in chylomicrons and extremely large VLDL mmol/l	1,32E-02 (7,25E-03-2,21E-02)	2,59E-02 (1,53E-02-4,26E-02)	4,33E-02
Phospholipids in chylomicrons and extremely large VLDL mmol/l	2,13E-03 (1,54E-03-3,96E-03)	3,94E-03 (2,53E-03-6,02E-03)	4,12E-02
Free cholesterol in chylomicrons and extremely large VLDL mmol/l	1,56E-03 (1,17E-03-2,74E-03)	2,90E-03 (1,71E-03-4,06E-03)	3,17E-02
Triglycerides in chylomicrons and extremely large VLDL mmol/l	7,28E-03 (2,52E-03-1,41E-02)	1,44E-02 (8,20E-03-2,87E-02)	4,33E-02
Concentration of very large VLDL particles mol/l	5,06E-10 (3,40E-10-8,77E-10)	9,86E-10 (5,53E-10-1,37E-09)	3,72E-02
Total lipids in very large VLDL mmol/l	5,00E-02 (3,46E-02-8,75E-02)	9,74E-02 (5,48E-02-1,35E-01)	4,12E-02
Phospholipids in very large VLDL mmol/l	9,55E-03 (6,63E-03-1,60E-02)	1,74E-02 (1,07E-02-2,35E-02)	3,60E-02
Triglycerides in very large VLDL mmol/l	2,91E-02 (2,08E-02-5,03E-02)	5,80E-02 (3,23E-02-8,41E-02)	3,50E-02
Concentration of large VLDL particles mol/l	3,13E-09 (2,12E-09-5,18E-09)	5,23E-09 (3,17E-09-7,83E-09)	4,33E-02
Total lipids in large VLDL mmol/l	1,88E-01 (1,27E-01-3,06E-01)	3,09E-01 (1,88E-01-4,57E-01)	4,33E-02
Phospholipids in large VLDL mmol/l	3,80E-02 (2,68E-02-6,02E-02)	6,10E-02 (3,90E-02-8,62E-02)	4,33E-02
Free cholesterol in large VLDL mmol/l	2,49E-02 (1,72E-02-3,99E-02)	3,93E-02 (2,49E-02-5,53E-02)	4,33E-02
Triglycerides in large VLDL mmol/l	1,01E-01 (6,52E-02-1,70E-01)	1,75E-01 (1,06E-01-2,62E-01)	4,12E-02
Mean diameter for VLDL particles nm	5,35E-01 (1,25E-01-1,02E+00)	1,26E+00 (6,38E-01-1,96E+00)	1,13E-03
Ratio of triglycerides to phosphoglycerides	1,97E-01 (1,18E-01-2,86E-01)	2,85E-01 (1,92E-01-3,74E-01)	4,33E-02
Estimated degree of unsaturation^⁎⁎^	-8,50E-03 (-4,45E-02-6,75E-03)	-7,25E-02 (-1,02E-01–4,33E-02)	2,76E-08
22:6, docosahexaenoic acid mmol/l^⁎⁎^	1,05E-01 (7,34E-02-1,38E-01)	2,30E-02 (1,25E-02-4,64E-02)	1,26E-10
Omega-3 fatty acids mmol/l^⁎⁎^	3,03E-01 (2,31E-01-3,84E-01)	1,03E-01 (5,56E-02-1,72E-01)	6,81E-10
Ratio of 22:6 docosahexaenoic acid to total fatty acids^⁎⁎^	3,05E-01 (3,60E-02-4,81E-01)	-2,77E-01 (-3,82E-01–1,61E-01)	3,15E-11
Ratio of omega-3 fatty acids to total fatty acids^⁎⁎^	8,90E-01 (1,08E-01-1,29E+00)	-5,94E-01 (-9,57E-01–3,01E-01)	3,15E-11
Ratio of polyunsaturated fatty acids to total fatty acids^⁎⁎^	-2,12E+00 (-3,47E+00–8,95E-01)	-4,78E+00 (-6,11E+00–3,03E+00)	1,79E-05
Ratio of monounsaturated fatty acids to total fatty acids^⁎⁎^	1,88E+00 (9,43E-01-3,00E+00)	3,36E+00 (2,57E+00-4,37E+00)	1,51E-04

*Mann Whitney U-test

^⁎⁎^Lipids reflecting the intake of fish oil

**Table 3:** b. Statistically significant differences in the change of metabolites from early to late pregnancy between the fish oil+probiotics group and the placebo+placebo in women without GDM. The values are expressed as median (IQR) concentration for the difference between early and late pregnancy.

	Fish oil+probiotics (*n* = 60)	Placebo+placebo (*n* = 64)	Fish oil+probiotics vs placebo+placebo
	Median (IQR)	Median (IQR)	BH-adjusted *P*-value*
Cholesterol esters to total lipids ratio in large VLDL	-6,10E-01 (-1,98E+00-8,50E-02)	-2,27E+00 (-3,71E+00–8,33E-01)	1,15E-02
Total cholesterol to total lipids ratio in medium VLDL	1,75E+00 (7,78E-01-3,99E+00)	1,10E-01 (-1,85E+00-1,99E+00)	1,15E-02
Cholesterol esters to total lipids ratio in medium VLDL	3,50E-01 (-7,78E-01-2,22E+00)	-1,16E+00 (-3,24E+00-4,45E-01)	3,94E-03
Triglycerides to total lipids ratio in medium VLDL	-2,11E+00 (-4,42E+00–7,68E-01)	-7,00E-02 (-2,23E+00-2,32E+00)	1,02E-02
Total cholesterol to total lipids ratio in small VLDL	2,77E+00 (8,45E-01-5,10E+00)	6,70E-01 (-2,07E+00-2,55E+00)	5,08E-03
Cholesterol esters to total lipids ratio in small VLDL	2,29E+00 (6,55E-01-4,18E+00)	2,00E-02 (-2,20E+00-2,07E+00)	4,92E-03
Triglycerides to total lipids ratio in small VLDL	-1,23E+00 (-3,64E+00-5,50E-01)	1,14E+00 (-1,27E+00-3,48E+00)	9,11E-03
Phospholipids to total lipids ratio in very small VLDL	-2,45E-01 (-8,85E-01-4,83E-01)	-9,50E-01 (-1,61E+00–1,63E-01)	3,79E-02
Total cholesterol to total lipids ratio in very small VLDL	-1,50E+00 (-2,74E+00-2,13E-01)	-2,86E+00 (-4,61E+00–1,65E+00)	2,36E-02
Free cholesterol to total lipids ratio in very small VLDL	6,50E-02 (-2,33E-01-3,88E-01)	-3,05E-01 (-7,95E-01-1,23E-01)	2,92E-02
Triglycerides to total lipids ratio in very small VLDL	1,45E+00 (-7,93E-01-4,04E+00)	4,05E+00 (2,02E+00-5,77E+00)	5,22E-03
Free cholesterol to total lipids ratio in IDL	-4,90E-01 (-9,40E-01–6,50E-02)	-1,27E+00 (-1,80E+00–6,70E-01)	5,34E-04
Total cholesterol to total lipids ratio in large LDL	1,28E+00 (-1,20E-01-2,41E+00)	1,45E-01 (-9,58E-01-1,50E+00)	3,79E-02
Phospholipids to total lipids ratio in medium LDL	-3,86E+00 (-5,02E+00–2,20E+00)	-2,20E+00 (-4,09E+00–8,48E-01)	2,05E-02
Total cholesterol to total lipids ratio in medium LDL	2,87E+00 (8,15E-01-4,34E+00)	8,55E-01 (-1,26E+00-2,74E+00)	9,60E-03
Cholesterol esters to total lipids ratio in medium LDL	5,28E+00 (3,31E+00-8,00E+00)	3,15E+00 (3,48E-01-6,11E+00)	1,81E-02
Phospholipids to total lipids ratio in small LDL	-4,84E+00 (-6,40E+00–2,98E+00)	-2,93E+00 (-5,45E+00–8,93E-01)	3,79E-02
Total cholesterol to total lipids ratio in small LDL	3,31E+00 (1,38E+00-5,26E+00)	9,15E-01 (-1,60E+00-3,51E+00)	9,60E-03
Cholesterol esters to total lipids ratio in small LDL	5,36E+00 (3,08E+00-7,84E+00)	2,45E+00 (-6,40E-01-6,16E+00)	1,92E-02
Triglycerides to total lipids ratio in small LDL	1,23E+00 (9,75E-02-2,17E+00)	2,16E+00 (1,29E+00-3,04E+00)	2,89E-02
Phospholipids to total lipids ratio in small HDL	-4,74E+00 (-6,21E+00–3,05E+00)	-2,54E+00 (-5,39E+00–4,95E-01)	4,34E-02
Total cholesterol to total lipids ratio in small HDL	2,62E+00 (1,18E+00-4,61E+00)	2,90E-01 (-2,70E+00-2,71E+00)	1,32E-02
Cholesterol esters to total lipids ratio in small HDL	2,92E+00 (1,30E+00-5,12E+00)	5,10E-01 (-2,57E+00-3,11E+00)	2,05E-02

*Mann Whitney U-test

Further evidence that the intervention effect was dependent on the GDM status was observed from the interaction analysis, in which significant interactions were found between GDM status and fish oil+probiotic and placeo+placebo in many lipids and those variables associated with the intake of fish oil, i.e. the DHA, ratio of DHA and MUFA to total fatty acids (Supplementary table 2) although after correcting for multiple testing, these interactions were no longer statistically significant (FDR>0•05, two-way analysis of variance). Nonetheless, the finding that the response to the intake of fish oil differed according to the GDM status was confirmed in the PCA analysis, (Supplemental Figure 6).

Secondly, in the fish oil group, there were a few differences detected in comparison to the placebo+placebo group, and again, in women without GDM, nine variables differed (FDR<0•05), e.g. the increase in median diameter of VLDL-particles increased less and free cholesterol to total lipids ratio in IDL decreased less in the fish oil+placebo group when compared to the placebo+placebo group (Table 4). When women with GDM were analysed, no differences were observed in any metabolites, between those in the fish oil+placebo as compared to the placebo+placebo group (Supplemental table 2). Similarly to the fish oil+probiotics group, depending their GDM status, the women responded differentially in their lipid values reflecting the intake of fish oil, as observed in the PCA (Supplemental figure 7).

**Table 4:** Statistically signifianct differences in the change of metabolites from early to late pregnancy between the fish oil+placebo and the placebo+placebo-group in women without GDM. The values are expressed as median (IQR) of the difference between early and late pregnancy concentrations.

	Fish oil+placebo (*n* = 62)	Placebo+placebo (*n* = 64)	Fish oil+placebo (*n* = 62) vs placebo+placebo (*n* = 64)
	Median (IQR)	Median (IQR)	BH-adjusted *P*-value*
Free cholesterol to total lipids ratio in IDL	-5,45E-01 (-1,10E+00–1,70E-01)	-1,27E+00 (-1,80E+00–6,70E-01)	4,43E-03
Mean diameter for VLDL particles nm	6,30E-01 (1,65E-01-1,21E+00)	1,26E+00 (6,38E-01-1,96E+00)	1,10E-02
Estimated degree of unsaturation^⁎⁎^	-2,50E-02 (-5,05E-02-1,50E-03)	-7,25E-02 (-1,02E-01–4,33E-02)	3,18E-07
22:6, docosahexaenoic acid mmol/l ^⁎⁎^	9,50E-02 (5,70E-02-1,32E-01)	2,30E-02 (1,25E-02-4,64E-02)	4,46E-08
Omega-3 fatty acids mmol/l^⁎⁎^	2,87E-01 (1,71E-01-3,81E-01)	1,03E-01 (5,56E-02-1,72E-01)	2,21E-07
Ratio of 22:6 docosahexaenoic acid to total fatty acids^⁎⁎^	1,60E-01 (-4,65E-02-3,89E-01)	-2,77E-01 (-3,82E-01–1,61E-01)	6,69E-11
Ratio of omega-3 fatty acids to total fatty acids^⁎⁎^	4,62E-01 (-1,23E-01-1,12E+00)	-5,94E-01 (-9,57E-01–3,01E-01)	9,94E-11
Ratio of polyunsaturated fatty acids to total fatty acids^⁎⁎^	-2,80E+00 (-4,08E+00–1,63E+00)	-4,78E+00 (-6,11E+00–3,03E+00)	1,02E-03
Ratio of monounsaturated fatty acids to total fatty acids^⁎⁎^	1,95E+00 (8,80E-01-3,37E+00)	3,36E+00 (2,57E+00-4,37E+00)	6,70E-04

*Mann Whitney u test

**Lipids reflecting the intake of fish oil

When evaluating the response of fish oil on those lipids reflecting the intake of fish oil fatty acids, we also analysed the changes without correcting for multiple testing. A distinct response to fish oil was observed according to the GDM status (Supplemental figure 8, Supplemental table 3). In healthy women, fish oil induced changes in nearly all of the lipids that are estimated to reflect the intake of fish oil (estimated degree of unsaturation, DHA, omega-3 fatty acids, ratio of DHA, omega-3, PUFA and MUFA to total lipids) both in the fish oil+placebo and the fish oil+probiotics groups when compared to the placebo+placebo group, which was not the case in women with GDM (Supplemental Figure 8, Supplemental table 3).

## 4 Discussion

Our findings show that dietary supplementation with fish oil and particularly the combination of fish oil and probiotics were able to modify the levels of serum lipids in pregnant women with overweight or obesity. Most notably, the effect was seen in the lipid contents of VLDL and LDL in women without GDM. When compared to the placebo+placebo group, i.e. the changes driven by pregnancy itself, the results indicated that the combination achieved potential benefits in terms of the long-term risk for developing metabolic disturbances.

The findings emerging from this study indicate that pregnant women with overweight or obesity may benefit from the supplementation with the combination of fish oil and probiotics. This is evident when comparing the changes in the ratios of various lipids to total lipids in lipoproteins. For example, in medium and small VLDL-particles, the ratio of triglycerides to total lipids decreased and the ratio of cholesterol increased in the fish oil+probiotics group as compared to the placebo+placebo group, in which the changes were less pronounced or in the opposite direction. In our previous studies, a higher ratio of triglycerides and a lower ratio of total cholesterol and cholesterol ester to lipids in VLDL-particles were associated with GDM [9,14] and in another study with the incidence of type 2 diabetes [30]. Further favorable alterations were observed in the ratios of cholesterols and phospholipids to total lipids in medium and small LDL-and small HDL-particles, i.e. particles known to be associated with cardiovascular diseases [31,32].

The changes in the ratio of various lipids in lipoproteins were evident when all the women were analysed, but interestingly, as the further analysis revealed, only in women without GDM. In addition, the increases were detected only in women without GDM in the concentration and in the lipid contents of larger sized VLDL-particles; these were lower in the fish oil+probiotics group when compared to the placebo+placebo group. Furthermore, the intake of fish oil was reflected in several fatty acids and their ratios, and again the influence was more evident in women without GDM. These results are of importance considering metabolic health as we have previously shown that higher concentrations of VLDL-particles, as well as their lipid content, are associated with GDM status [11,19]. Furthermore, previous studies in non-pregnant populations, have reported associations between higher triglyceride and cholesterol concentrations in VLDL-particles, PUFA and DHA and serious illnesses e.g. cardiovascular diseases (myocardial infraction, ischemic stroke) [33] and incident type 2 diabetes [30]. The approach of using food supplements from early pregnancy onwards may thus represent a feasible way for improving metabolism during pregnancy with potential long-term health benefits.

Our findings suggest that the intake of a combination of fish oil and probiotics may benefit lipid metabolism particularly in women without GDM. As compliance to the supplementation was similar in both groups, the results indicate that women with GDM are less responsive to dietary supplementation. One likely reason for the lack of response to the intervention in the measured metabolites in women with GDM may arise from the increased metabolic burden in these women, as we and others have previously observed [19,34].

Contrary to the potentially favorable alterations observed in VLDL-particles, we observed that the ratio of free cholesterol in women without GDM increased to a greater extent whereas the ratio of cholesterol ester in large VLDL decreased in all women and in women without GDM less in the fish oil+probiotics group as compared to those in the placebo+placebo group. Furthermore, in very small VLDL-particles, the total cholesterol amount decreased, but less so in the fish oil+probiotics group when compared to the placebo+placebo group. These findings might indicate that the intervention had an unfavorable impact also noted in our previous study where a higher ratio of free cholesterol and a lower ratio of cholesterol esters in larger VLDL were related to GDM [11]. Furthermore, a lower ratio of total cholesterol to total lipids in very small VDLD has been associated with a higher risk of incident type 2 diabetes [30] and GDM [19]. The origin of the distinct response of different sized lipoproteins may arise from the different roles of fish oil fatty acids in lipoprotein handling pathways [35] in which the lipoprotein particles are metabolized [31]. More studies will be needed to clarify the differential response of different sized lipoproteins to the intervention and their outcomes over the long term.

In our study, fewer changes in the metabolites took place in the fish oil+placebo than with the combination of fish oil+probiotics, suggesting a synergistic impact of fish oil and probiotics on maternal metabolism. Our findings detailing the intake of fish oil and probiotics as a combination are new and potentially represent a feasible approach to modifying maternal metabolism. Previous evidence using metabolomics in non-pregnant adults found signs of a metabolic shift due to the consumption of probiotics or fish oil, when probiotics and fish oil were supplemented individually [15], [16], [17], [18]. The mechanisms behind the metabolic effect of probiotics in augmenting the effect of fish oil have still to be defined, but one explanation possibly arises from the capability of probiotics and fish oil to regulate soluble (s)CD4, a pattern recognition receptor, which is involved not only in antimicrobial host defence, but also in lipid transfer [36]. This may lead to the enhanced transport of fatty acids including PUFA in blood, and a subsequent increase in the production of PUFA-derived immunomodulators, such as eicosanoids, as previously suggested [37]. Both fish oil and probiotics have also been shown to strengthen the intestinal epithelial barrier, as reviewed by Mokkala et al. [38] and also in this same study population, to modulate the composition of the gut microbiota [39]; these mechanisms may be involved behind the synergistics effects observed. All in all, the combination of fish oil and probiotics, particularly in women without GDM, induced mostly favorable alterations in more metabolites than fish oil alone; we hope that this will trigger an interest towards the use of fish oil and probiotics as a combination to modify maternal metabolism, although further research is warranted to confirm the findings.

This study also illustrates the physiological changes occurring in serum metabolites in overweight and obese pregnant women. The traditional analysis methods for lipids include assays of serum triglycerides and various measures of cholesterol. Here, we undertook a robust metabolomics approach, which provided information on more than 200 metabolites with detailed data on various lipids, which we consider as an evident strength of this study. In this group of women with overweight or obesity, nearly all of the lipids that we measured increased in the placebo+placebo group; these represent the pregnancy induced alterations. In addition, several increases and decreases were observed in the levels of certain amino acids, and the low grade inflammation marker, GlycA increased, pointing to the presence of increased inflammation towards the end of pregnancy. The detected pregnancy driven alterations, are mostly in line with a previous study investigating normal weight pregnant women [40]. Our sample was similar to the general population of pregnant women in Finland with regard to maternal age and delivery parameters, although it was slightly different with regard to primiparity (48% in our sample vs. 58% in perinatal statistics) [41,42].

### 4.1 Caveats and limitations

It is noteworthy that the number of women with GDM was smaller than that of women without this disorder, and we also applied robust statistical methods, e.g. corrected for multiple testing and conducted a post hoc analysis, which may have decreased the statistical power to detect the possible differences, calling for confirmatory studies. Further, no power calculations were performed for these prespecified metabolites that were secondary outcomes of the study. It is possible that statistically significant changes in the metabolites could be detected with a larger number of samples, for example, due either to the intervention or the GDM status of the women. The women in our study were at a high risk for developing metabolic complication during pregnancy and postpartum, thus an investigation of the influence of dietary supplementation with probiotics/fish oil may provide a novel way to improve the metabolic profile of these women. However, one important topic for research would be whether the intervention would exert favorable effects in normal weight pregnant women. These findings apply to the specific probiotics strains used in this study and it is not known whether similar results can be obtained with other strains. Further, the data on clinical characteristics was collected by questionnaires, which introduces a selfreporting bias.

To conclude, we have demonstrated that the intake of fish oil and probiotics, *L. rhamnosus HN001* and *B. animalis ssp. lactis 420,* delivered as a combination may induce beneficial alterations in lipid metabolism in overweight or obese pregnant women with the response being influenced by the GDM status, i.e. the women without GDM showed more distinct changes in several lipids as compared to placebo+placebo, whereas in women with GDM, no such differences were observed. Our findings provide new information on the effects on serum lipids obtained following a supplementation with a combination of fish oil and probiotics, although considering the study limitations, it is evident that confirmatory studies are needed.

## Contributors

K.L and K.M conceptualisation. K.L supervision, administration and funding acquisition. K.M data curation. N.H, E.K, K.M investigation. K.M and T.V formal analysis and data verification, L.L contributed to formal analysis. K.M writing original draft, KM and K.L review and editing. All authors had full access to all data in the study and accept responsibility to submit for publication. All authors read and approved the final version of the manuscript.

## Data sharing statement

The datasets analyzed during the current study are available as deposited in Zenodo (Supplemental tables 1, 2 and 3: https://doi.org/10.5281/zenodo.4898766). The individual participant's data are not available due to the fact that they contain information that could compromise the privacy of our research participants

## Declaration of Competing Interest

None.

## Appendix Supplementary materials

Image, application 1

Image, application 2

Image, application 3
