# Supplementary material for: Impact of combined consumption of fish oil and probiotics on the serum metabolome in pregnant women with overweight or obesity
Source: eBioMedicine. 2021 Oct 30;73:103655. doi: 10.1016/j.ebiom.2021.103655 (PMC8577343; doi:10.1016/j.ebiom.2021.103655)
Supplement: Supplementary file 1 [file mmc1.docx]

Caption for supplementary material

Supplemental figure 1. Flow chart of the study.

Supplemental figure 2. Partial Least Squares (PLS) discriminant analysis for the seven lipid metabolites used for the PCA in Fig. 1. PLS scores (Fish Oil includes fish oil+placebo and fish oil+probiotics groups and No Fish Oil includes probiotics+placebo and placebo+placebo groups).

Supplemental figure 3. Partial Least Squares (PLS) discriminant analysis for the seven lipid metabolites used for the PCA in Fig. 1: PLS component loadings.

Supplemental figure 4. Workflow of the study.

Supplemental figure 5a-e. Metabolites (n=35) with statistically significant differences (Mann-Whitney U-test with post hoc Bonferroni corrections) in the changes between the fish oil+probiotics group and the placebo+placebo group. Absolute changes are presented. Blue: fish oil+probiotics, red:probiotics+placebo, grey: fish oil+placebo, orange: placebo+placebo.

Supplemental figure 6. PCA of the lipids that reflect the intake of fish oil in fish oil+probiotics-group in women without (blue) and with GDM (red).

Supplemental figure 7. PCA of the lipids that reflect the intake of fish oil in the fish oil+placebo -group in women without (blue) and with GDM (red).

Supplemental figure 8 Differences between intervention and placebo groups in women without or with GDM. * denote statistically significant (Kruskall-Wallis test followed by Bonferroni post hoc- test) difference in the change when compared to placebo group.

Supplemental table 1. Early and late pregnancy serum metabolites (median+IQR) and change from early to late pregnancy in metabolites in all women.

Supplemental table 2. Early and late pregnancy serum metabolites (median+IQR) and change from early to late pregnancy in metabolites in women with or without GDM.

Supplemental table 3. Response of fish oil on the lipids reflecting the intake of fish oil fatty acids.

The supplemental tables are available through Zenodo: <https://doi.org/10.5281/zenodo.4898766>
