# Supplementary material for: Impact of combined consumption of fish oil and probiotics on the serum metabolome in pregnant women with overweight or obesity
Source: eBioMedicine. 2021 Oct 30;73:103655. doi: 10.1016/j.ebiom.2021.103655 (PMC8577343; doi:10.1016/j.ebiom.2021.103655)
Supplement: Supplementary file 3 [file mmc3.pdf]

Supplemental figures

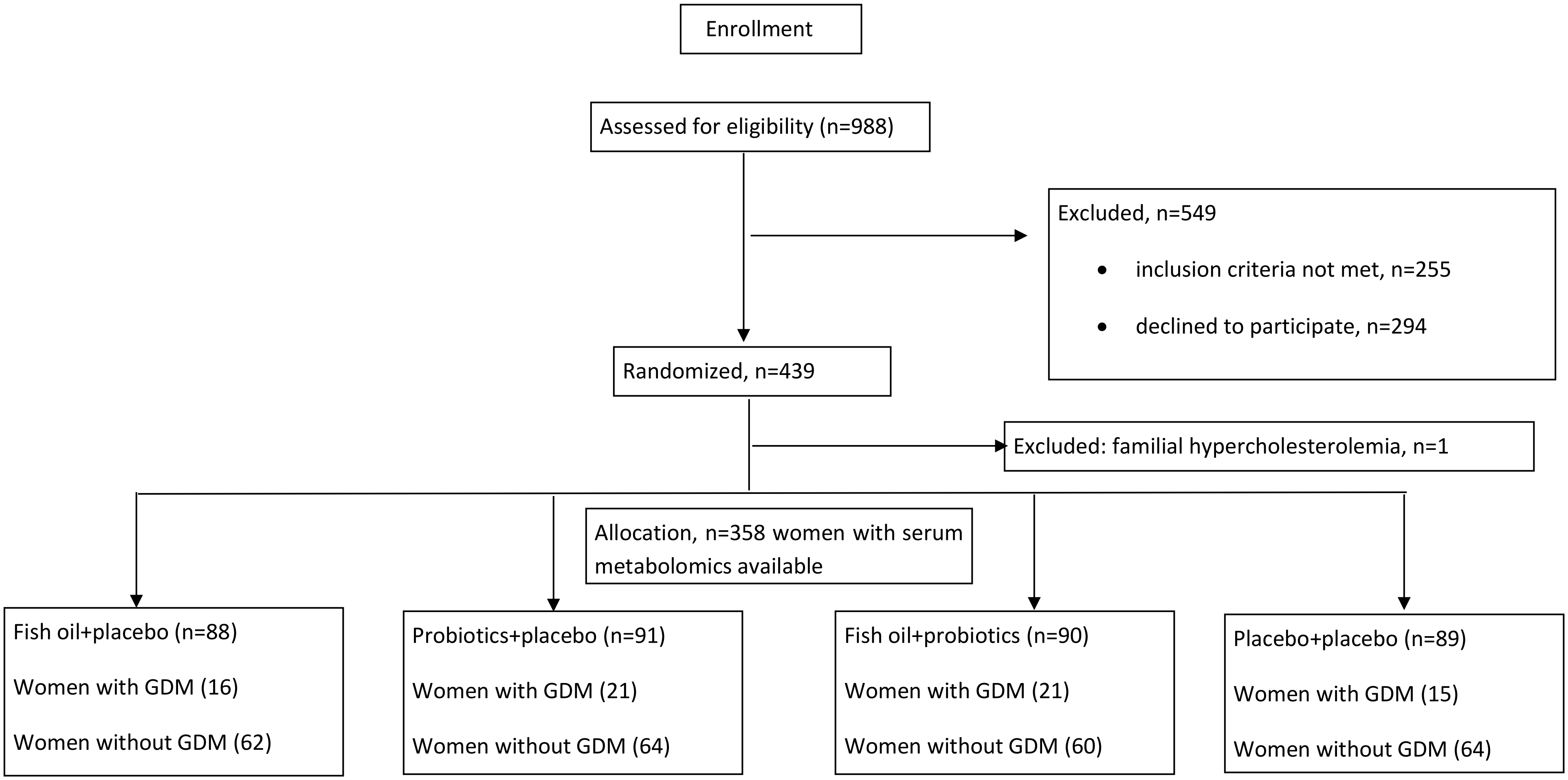

Supplemental figure 1. Flow chart.

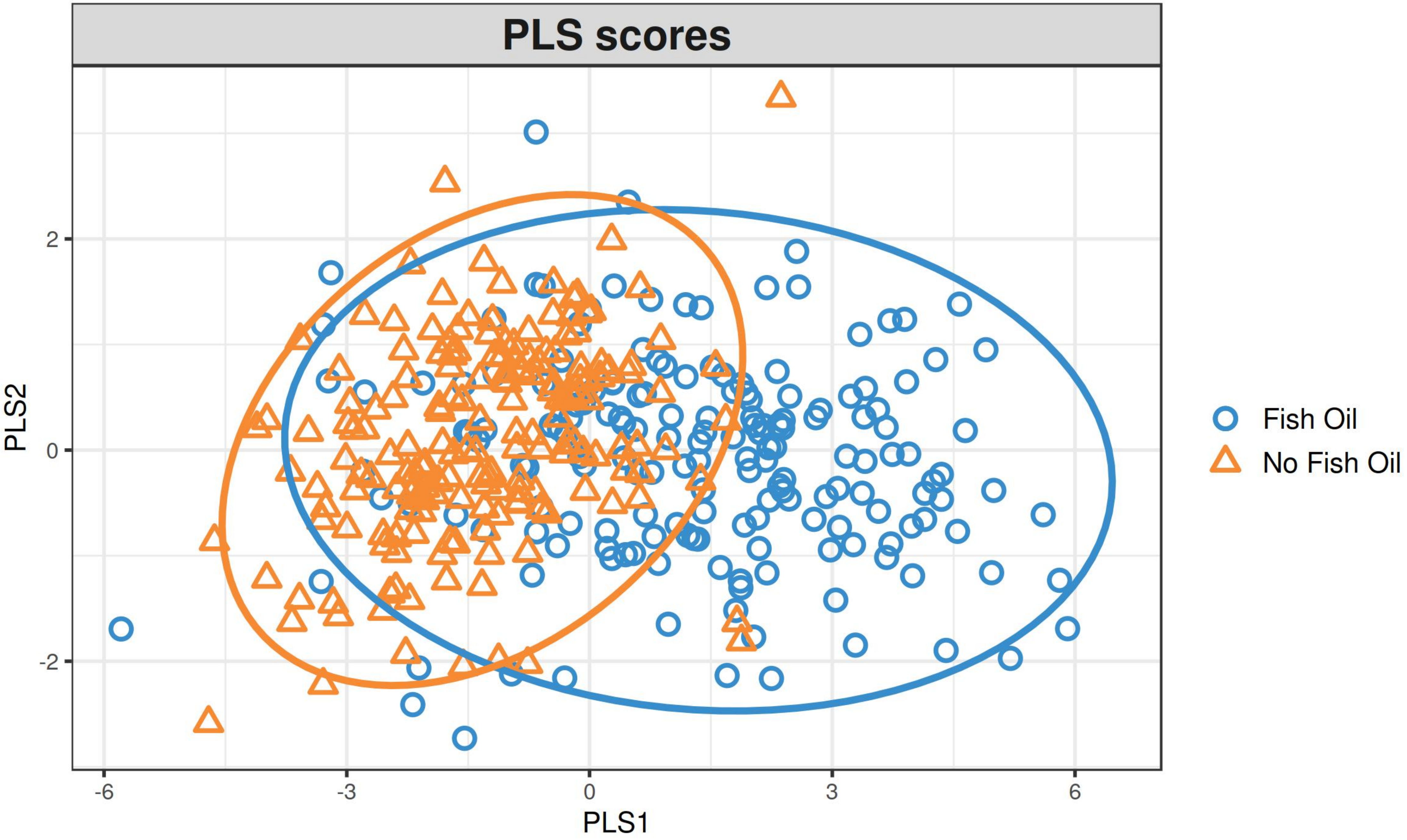

Supplemental figure 2. Partial Least Squares (PLS) discriminant analysis for the seven lipid metabolites used for the PCA in Fig. 1. PLS scores (Fish Oil includes fish oil+placebo and fish oil+probiotics groups and No Fish Oil includes probiotics+placebo and placebo+placebo groups).

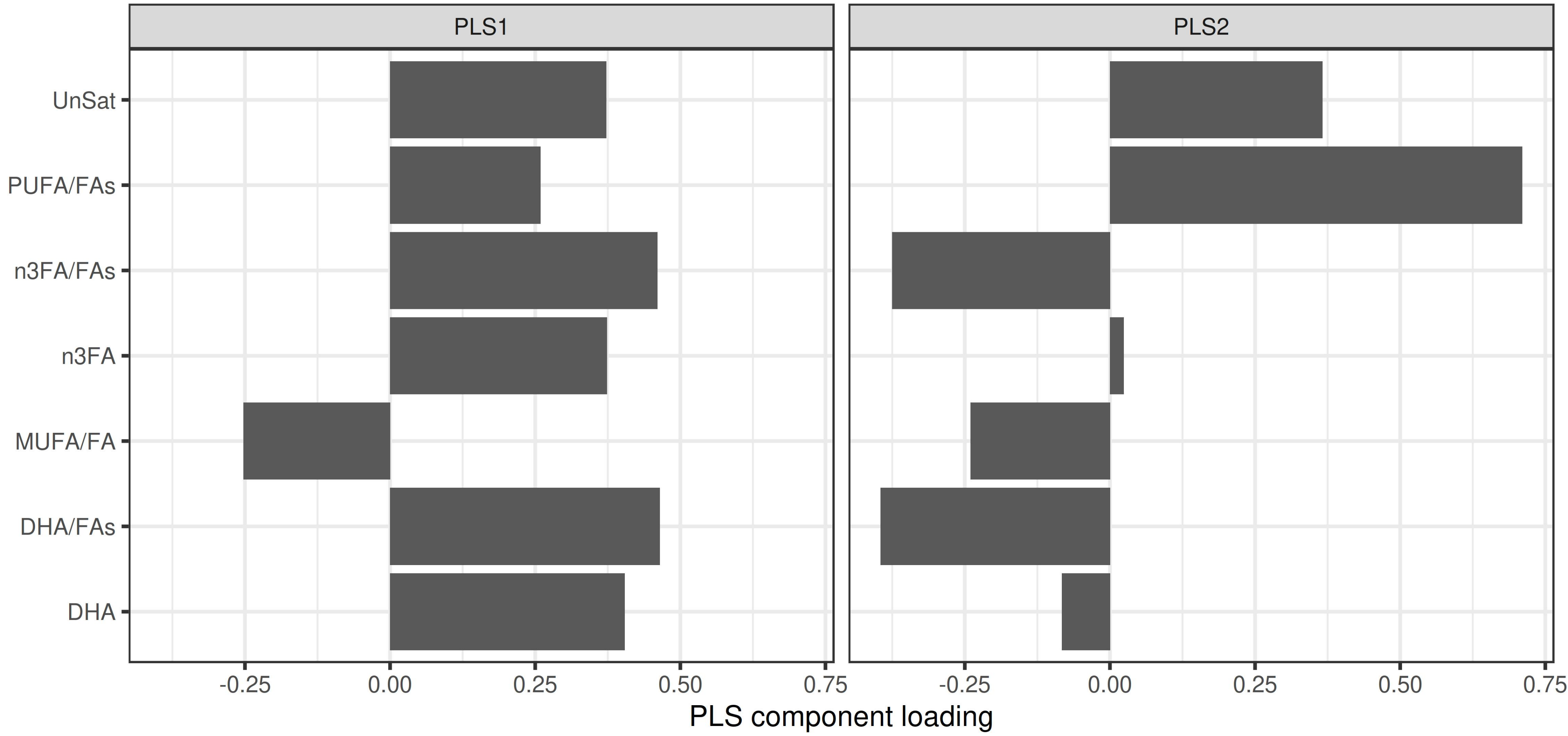

Supplemental figure 3. Partial Least Squares (PLS) discriminant analysis for the seven lipid metabolites used for the PCA in Fig. 1: PLS component loadings.

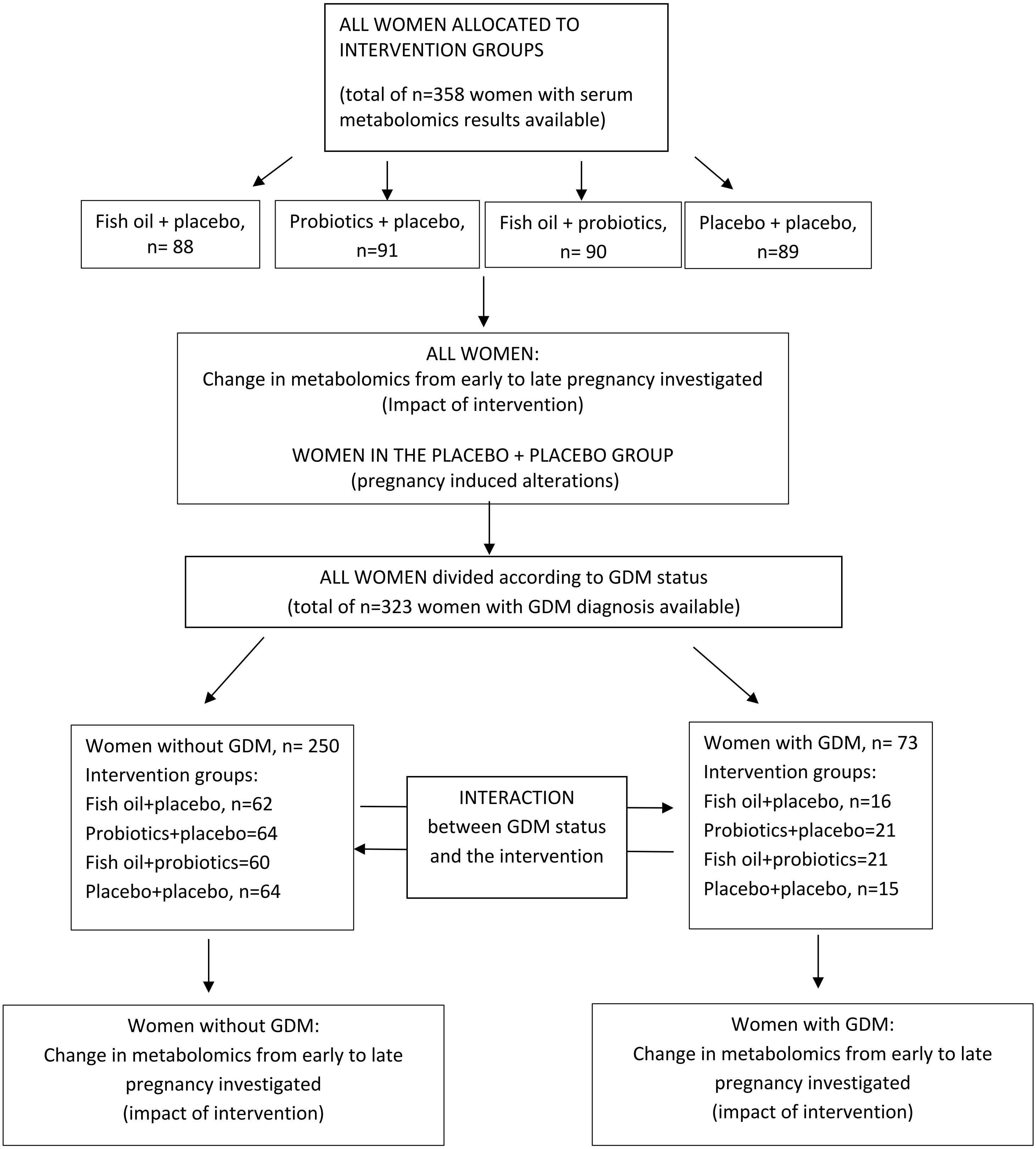

Supplemental figure 4. Workflow of the study.

Supplemental figures

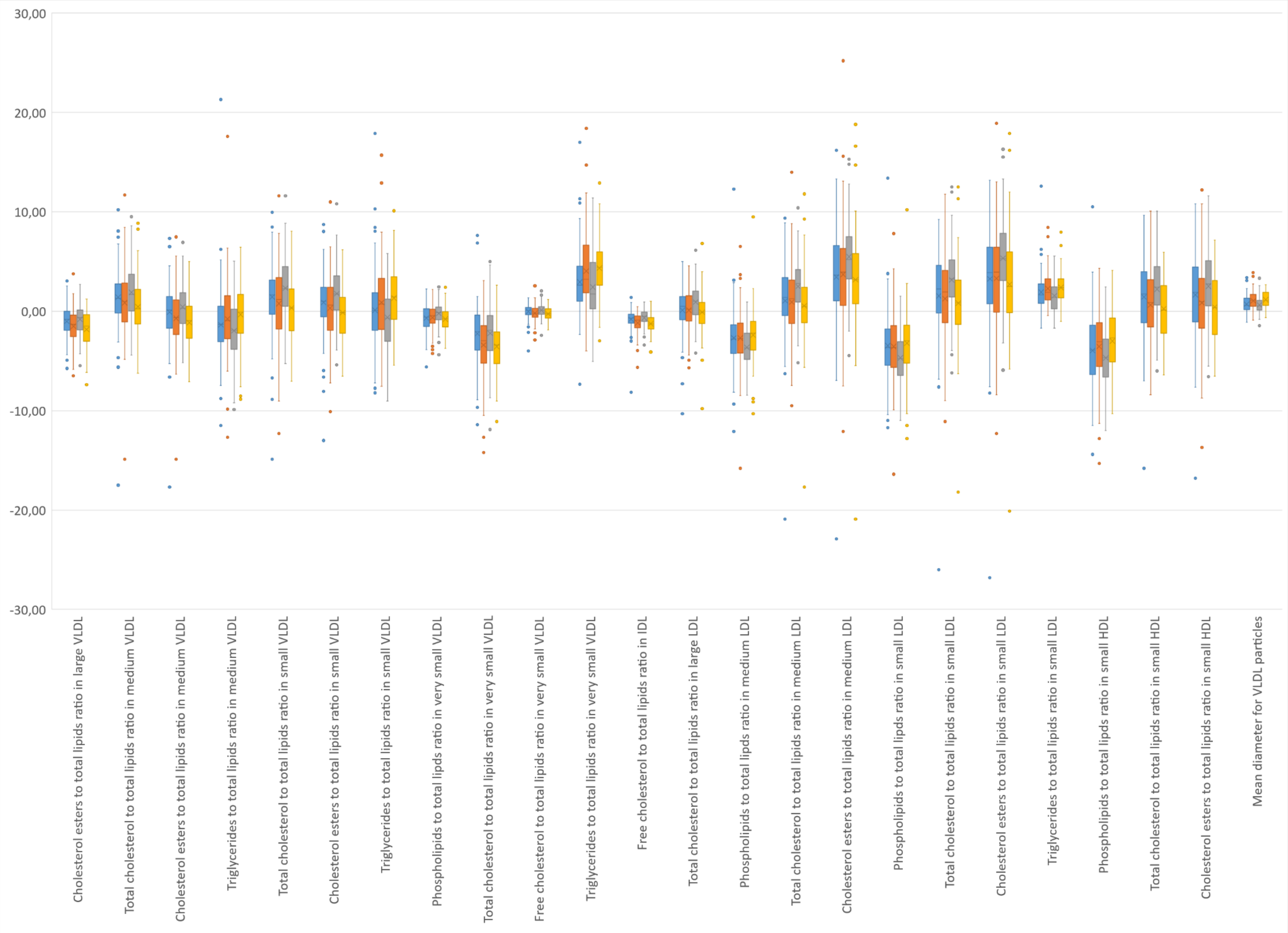

Fig 5a

Supplemental figures

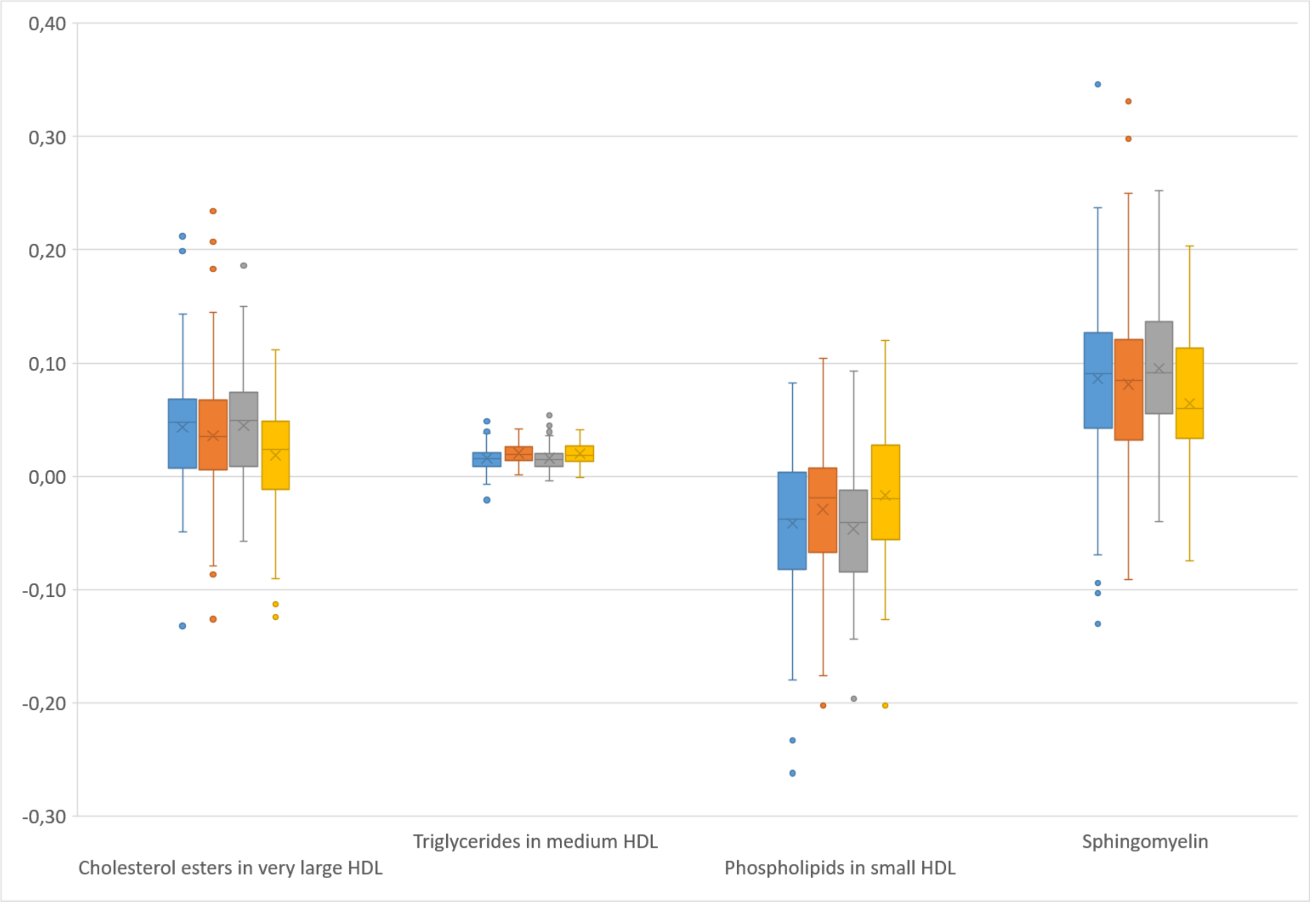

Fig 5b

Supplemental figures

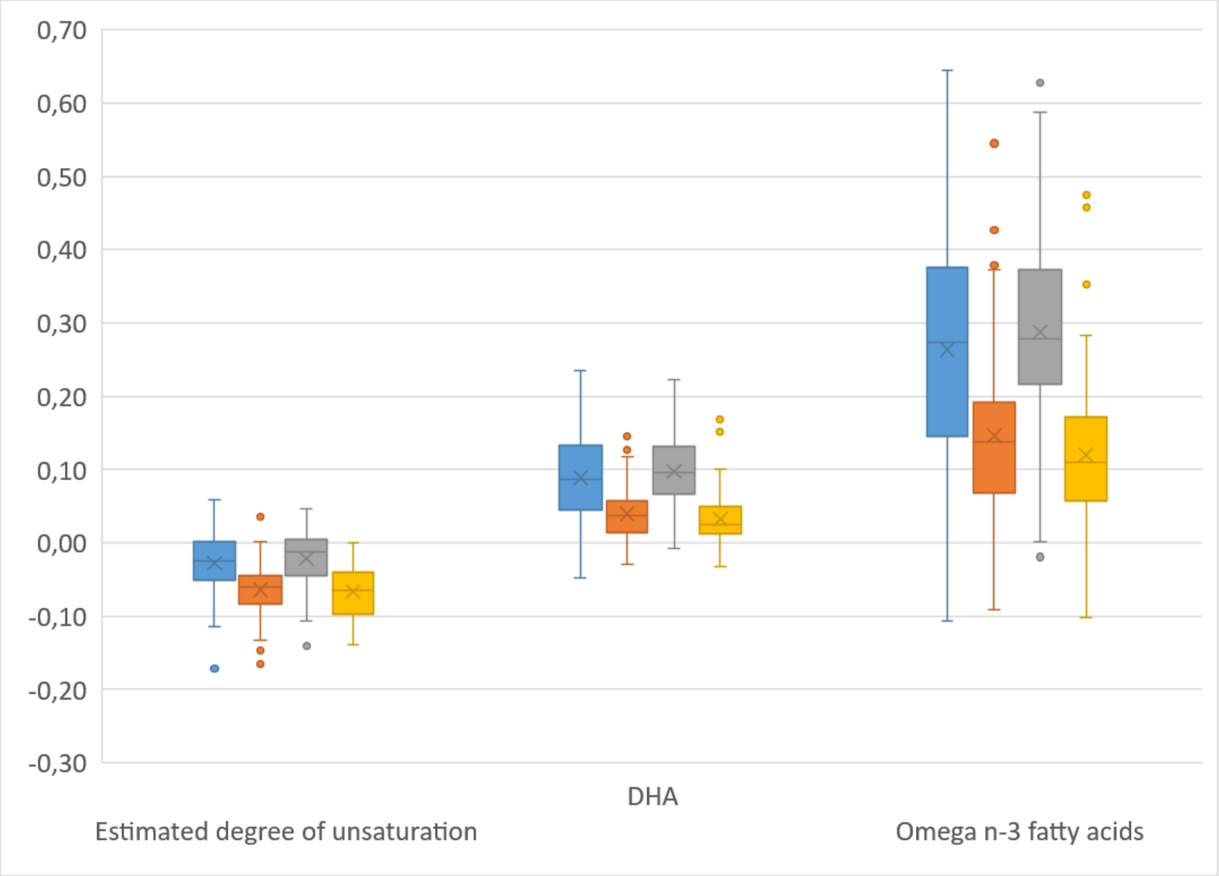

Fig 5c

Supplemental figures

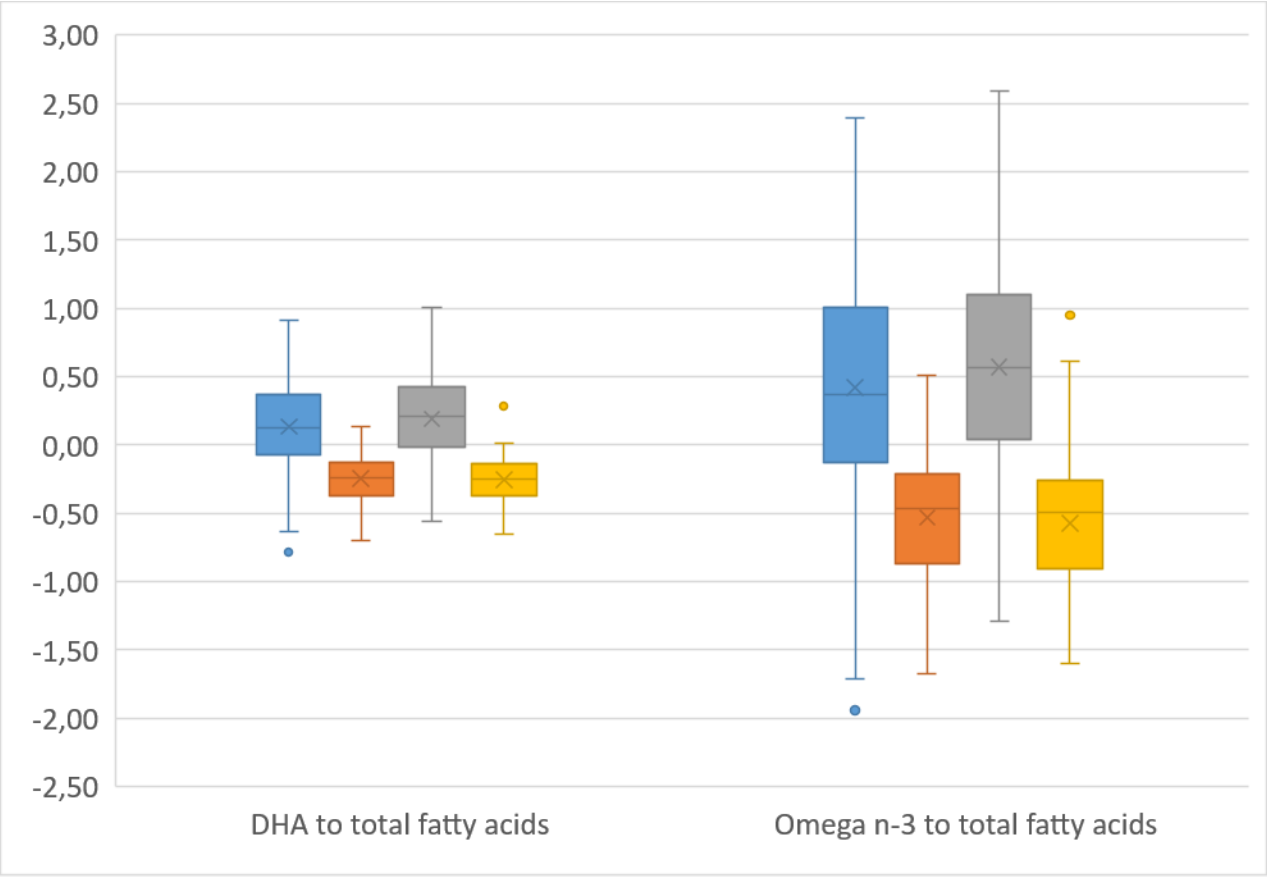

Fig 5d

Supplemental figures

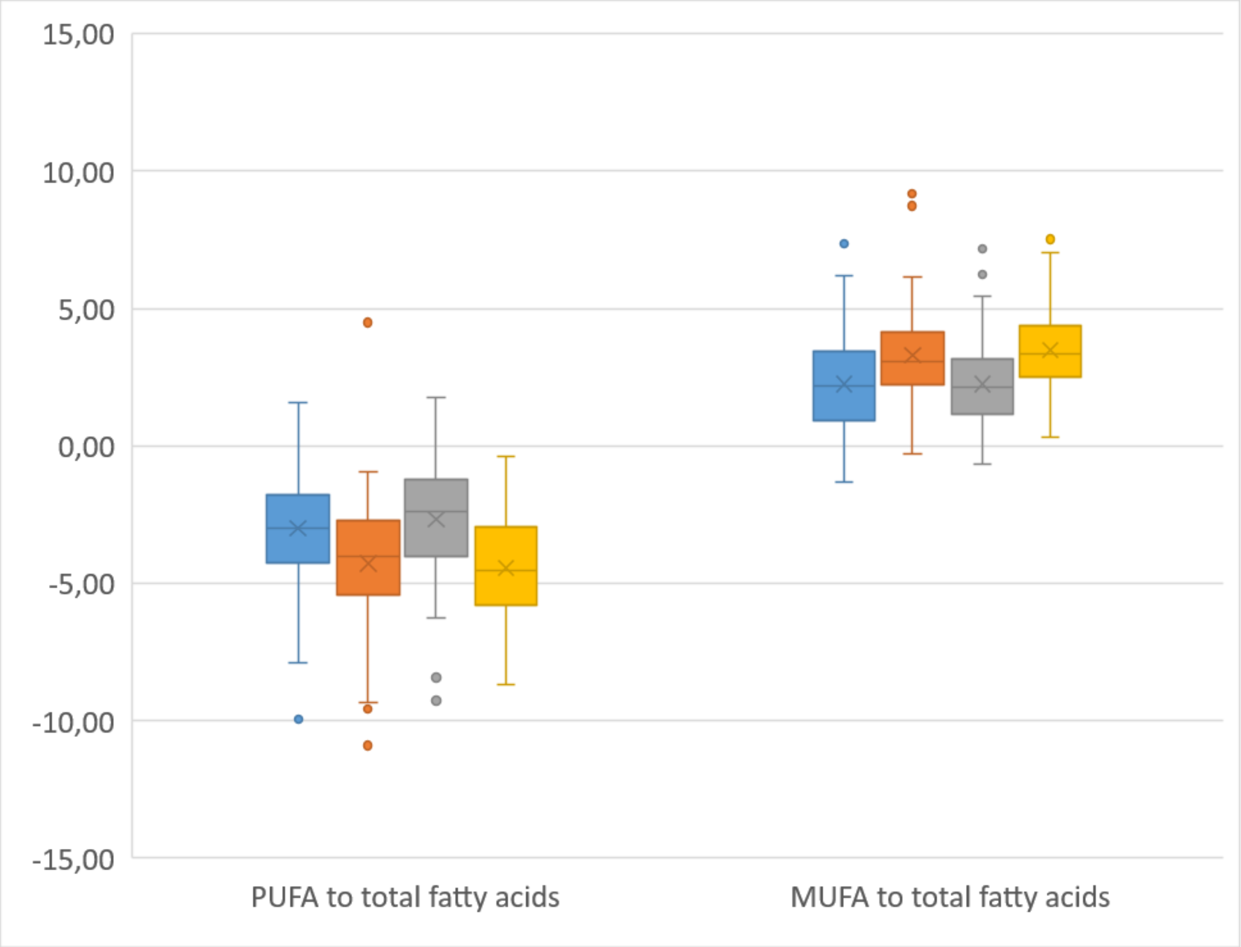

Fig 5e

Supplemental figure 5. Metabolites (n=35) with statistically significant differences (Mann Whitney test with post hoc Bonferroni corrections) in the changes between the fish oil+probiotics group and the placebo+placebo group. Absolute changes are presented. Blue: fish oil+probiotics, red:probiotics+placebo, gery: fish oil+placebo, orange: placebo+placebo

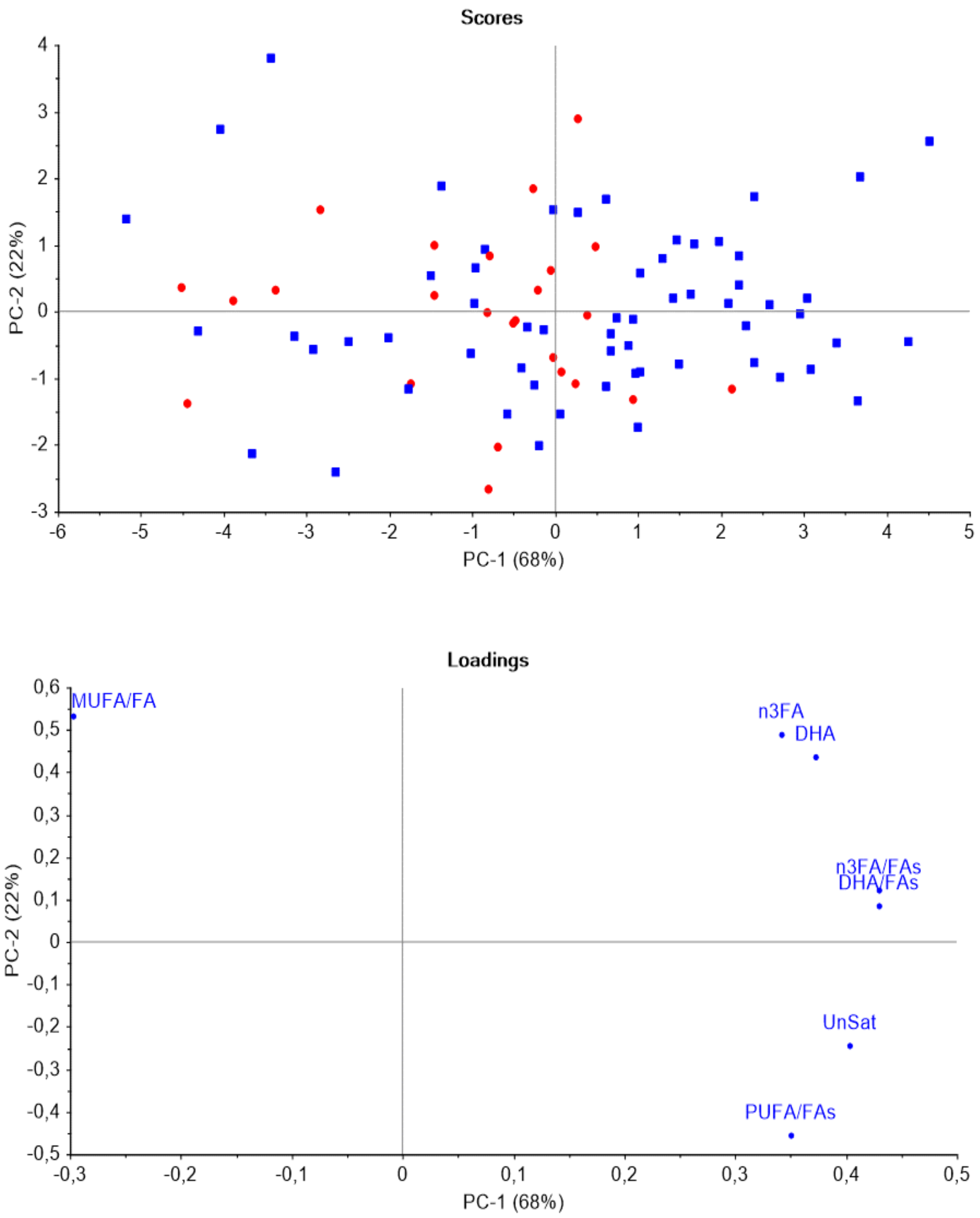

Supplemental figure 6. PCA of the lipids that reflect the intake of fish oil in fish oil+probiotics-group in women without (blue) and with GDM (red).

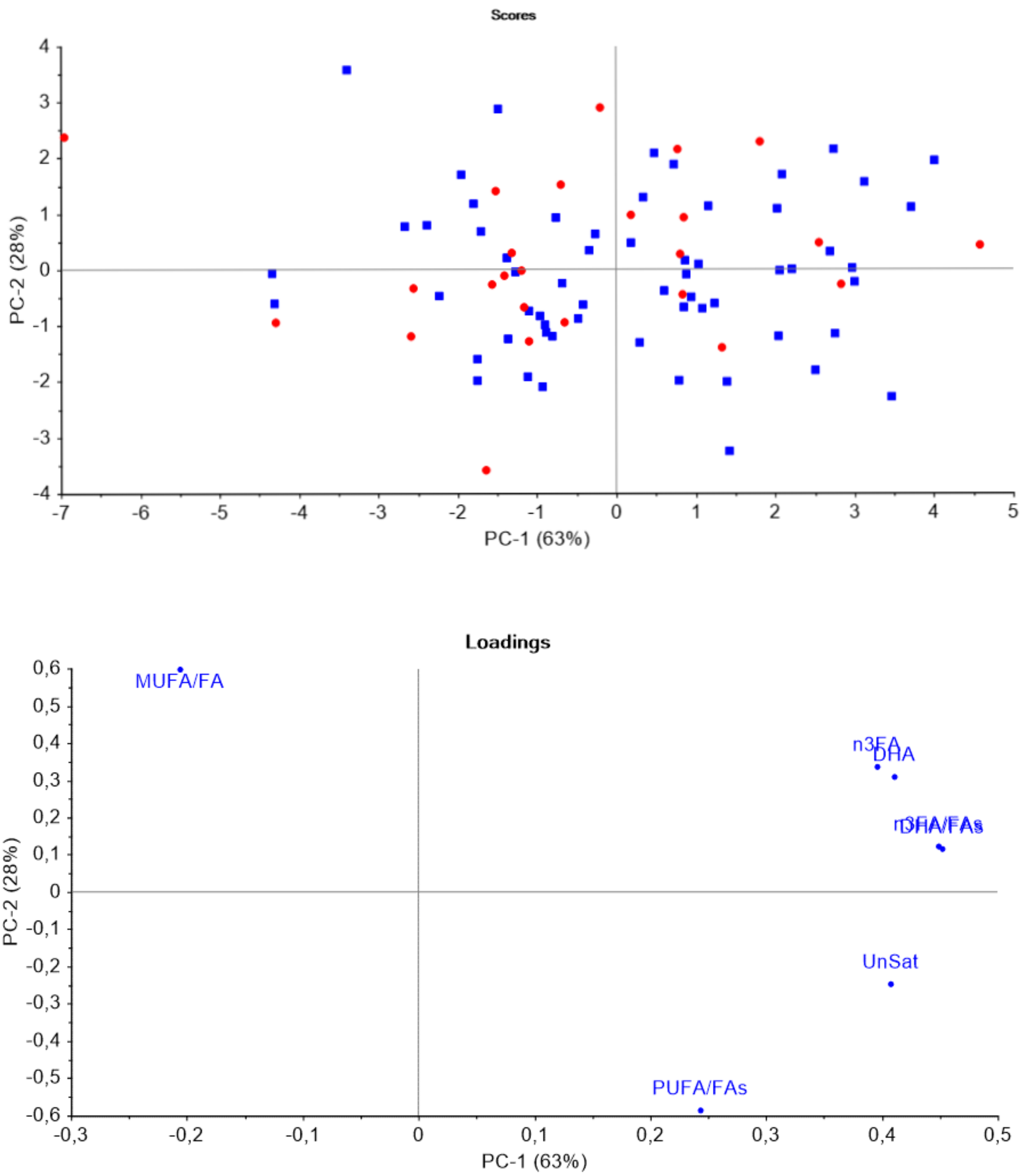

Supplemental figure 7. PCA of the lipids that reflect the intake of fish oil in the fish oil+placebo -group in women without (blue) and with GDM (red).

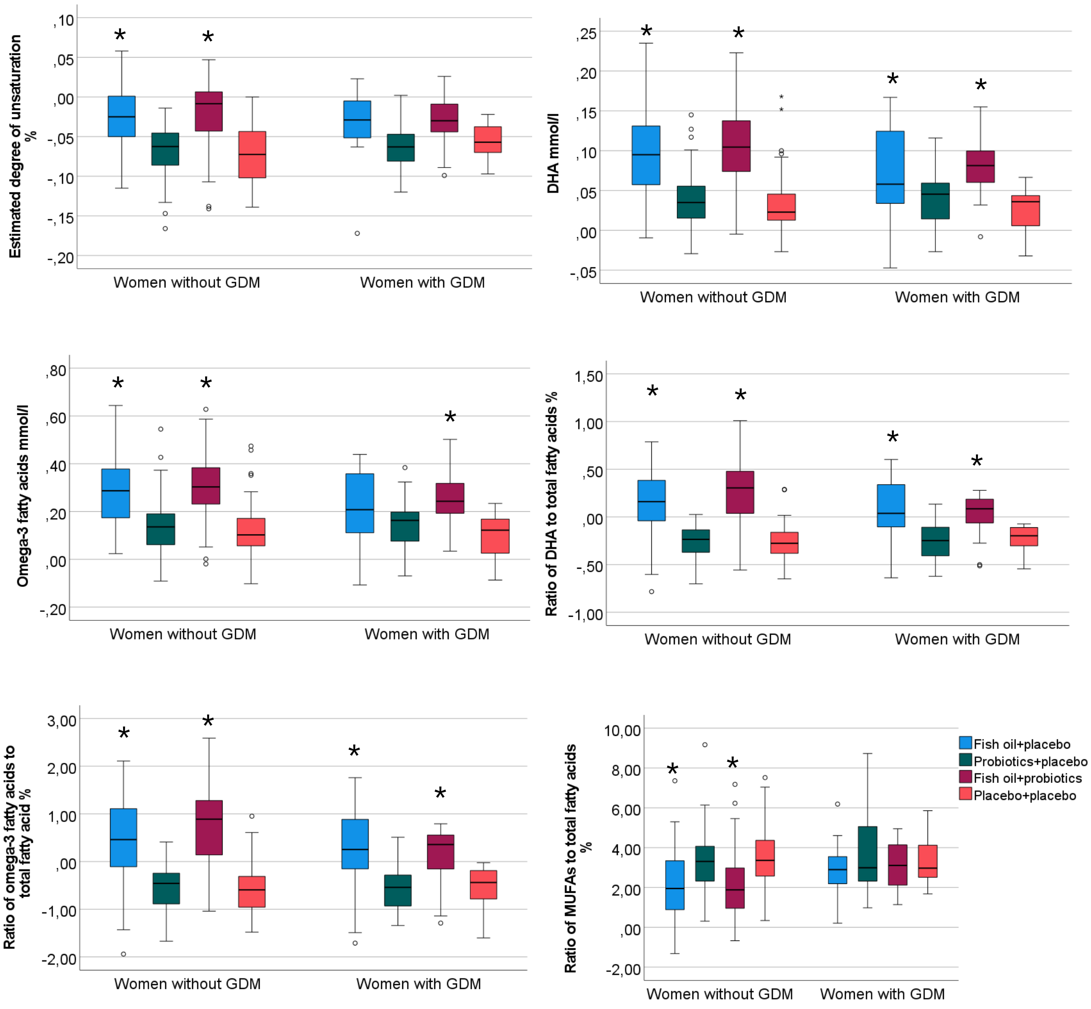

Supplemental figure 8. Differences between intervention and placebo groups in women without and with GDM. * denote statistically significant (Kruskall-Wallis test followed by Bonferroni post hoc-test) difference in change when compared to placebo group.
